# Individualized model for predicting pathological complete response to neoadjuvant chemotherapy in patients with breast cancer: A multicenter study

**DOI:** 10.3389/fendo.2022.955250

**Published:** 2022-08-17

**Authors:** Bei Qian, Jing Yang, Jun Zhou, Longqing Hu, Shoupeng Zhang, Min Ren, Xincai Qu

**Affiliations:** ^1^ Department of Thyroid and Breast Surgery, Union Hospital, Tongji Medical College, Huazhong University of Science and Technology, Wuhan, China; ^2^ Department of Breast Surgery, Department of General Surgery, First Affiliated Hospital of Anhui Medical University, Hefei, China

**Keywords:** breast cancer, BC, neoadjuvant chemotherapy, NACT, pathological complete response, pCR, clinicopathological features, nomogram

## Abstract

**Background:**

Pathological complete response (pCR) is considered a surrogate for favorable survival in breast cancer (BC) patients treated with neoadjuvant chemotherapy (NACT), which is the goal of NACT. This study aimed to develop and validate a nomogram for predicting the pCR probability of BC patients after NACT based on the clinicopathological features.

**Methods:**

A retrospective analysis of 527 BC patients treated with NACT between January 2018 and December 2021 from two institutions was conducted. Univariate and multivariate logistic regression analyses were performed to select the most useful predictors from the training cohort (n = 225), and then a nomogram model was developed. The performance of the nomogram was evaluated with respect to its discrimination, calibration, and clinical usefulness. Internal validation and external validation were performed in an independent validation cohort of 96 and 205 consecutive BC patients, respectively.

**Results:**

Among the 18 clinicopathological features, five variables were selected to develop the prediction model, including age, American Joint Committee on Cancer (AJCC) T stage, Ki67 index before NACT, human epidermal growth factor receptor 2 (HER2), and hormone receptor (HR) status. The model showed good discrimination with an area under the receiver operating characteristic curve (AUC) of 0.825 (95% CI, 0.772 to 0.878) in the training cohort, and 0.755 (95% CI, 0.658 to 0.851) and 0.79 (95% CI, 0.724 to 0.856) in the internal and external validation cohorts, respectively. The calibration curve presented good agreement between prediction by nomogram and actual observation, and decision curve analysis (DCA) indicated that the nomogram had good net benefits in clinical scenarios.

**Conclusion:**

This study constructed a validated nomogram based on age, AJCC T stage, Ki67 index before NACT, HER2, and HR status, which could be non-invasively applied to personalize the prediction of pCR in BC patients treated with NACT.

## Introduction

Breast cancer (BC) has become a malignant tumor with the largest number of new cases and deaths in women worldwide, posing a serious threat to women’s health (1). Neoadjuvant chemotherapy (NACT) has been widely used and has become the standard treatment for locally advanced BC (2). It not only can downstage the tumor before surgery and potentially convert an inoperable tumor to be resectable or convert from complete mastectomy to lumpectomy but also can enable the evaluation *in vivo* for sensitivity to different treatment regimens (3, 4). The pathological response of the primary breast tumor and axillary lymph nodes (LNs) after NACT reflects the sensitivity of the tumor to chemotherapy, which is also significantly associated with the prognosis. Pathological complete response (pCR) includes total pCR with no residual invasive tumor in the breast and LNs, and breast pCR with no residual invasive tumor in the breast only, regardless of the LNs (5, 6). It has been proposed that pCR to the NACT for BC patients is an early surrogate end-point for the prediction of disease-free survival (DFS) and overall survival (OS) (6–8).

However, not all BC patients treated with NACT can achieve pCR. For patients who do not achieve pCR, NACT may cause significant toxicity to the body, which may worsen their prognosis while increasing the cost of treatment (9). Moreover, identification of the pCR prior to surgery can facilitate adjustment of the optimal surgical strategy, especially for the management of the axilla (3). Currently, histopathological examination of the surgically resected specimens remains the gold standard for assessing pathological response after NACT. Although physical examination (PE) and conventional breast imaging such as magnetic resonance imaging (MRI) and ultrasound (US) are the main means to assess the pathological response after NACT, they are not efficient, and their accuracy is not always stable (10). Therefore, it is of great clinical value and significance to develop a safe, efficient, and non-invasive method for personalized prediction of pCR probabilities in BC patients treated with NACT both before NAC and before surgery.

The nomogram was used to assign scores to the level of each factor in the model according to the contribution to the outcome variable (regression coefficient). Then, a function conversion between the total score and the probability of the outcome event was performed, and the predicted probability of a certain clinical outcome was calculated. The nomogram is considered to be a reliable tool to predict the prognosis of cancer patients (11). However, nomograms based on multicenter and with relatively large samples are rarely reported. Thus, this study aims to develop and validate a nomogram for predicting pCR probability of BC patients after NACT based on the clinicopathological features.

## Materials and methods

### Data source and patient selection

This study retrospectively reviewed the data of BC patients treated with NACT at Union Hospital, Tongji Medical College, Huazhong University of Science and Technology, between January 2018 and December 2021. The exclusion criteria in the study were as follows: 1) vital organ was damaged; 2) important clinical parameters were absent or unavailable. A total of 325 BC patients who met the inclusion criteria were included in the primary cohort. Meanwhile, 205 BC patients treated with NACT from January 2018 through December 2021 at the First Affiliated Hospital of Anhui Medical University were screened as an independent external validation cohort. The extracted variables of eligible cases included age, menstrual status, height, weight, chemotherapy regimen, whether chemotherapy is completed (yes or no), and parameters before the start of NACT including peripheral blood albumin level, carbohydrate antigen 125 (CA125), carbohydrate antigen 199 (CA199), carcinoembryonic antigen (CEA), carbohydrate antigen 153 (CA153) levels, platelet count, neutrophil count, lymphocyte count, tumor size and presence of skin or chest wall invasion (yes or no), axillary LN status (positive or negative), Ki67 index, human epidermal growth factor receptor 2 (HER2) status (positive or negative), hormone receptor (HR) status (including estrogen receptor (ER) and progesterone receptor (PR); positive or negative), histological grade (I–III), and pathology-based Miller–Payne (MP) grade (G1–G5) after NACT.

### Pathological assessment

Before NACT, all patients were pathologically confirmed by core needle biopsy (CNB) of the primary breast tumor. All BC biopsy and surgical specimens were processed for histopathology and immunohistochemistry (IHC) and evaluated by two independent pathologists. ER or PR positivity was determined by IHC of at least 1% of the infiltrating tumor cells positive. HER2 positivity was defined as 3+ measured by IHC and/or positive HER2 gene amplification by fluorescence *in situ* hybridization (FISH) detection; otherwise, they were deemed to be negative. Tumor cell nuclei with a positive background immunostaining score greater than 20% were considered high expressing, and less than 20% were considered low expressing, which was used to discriminate between Luminal A and Luminal B subtypes (12). According to the guideline of the Nottingham Grading System (13), breast tumors were classified into corresponding histological grades. Tumor molecular subtypes were classified as Luminal A, Luminal B/HER2-positive, Luminal B/HER2-negative, HER2-enriched, and triple-negative, based on the IHC and FISH results. The MP grading system (G1–G5) was applied to grade the pathological response of the primary breast tumor after NACT. Grade 5 (G5) indicates no invasive cancer cells in the tumor bed at surgical resection regardless of node status, which was defined as pCR. Correspondingly, pathological reactions classified as grades 1–4 (G1–G4) were considered non-pCR.

### Variable recode

The cutoff value for the age group was set at 35 years according to the definition of young BC (14). PLR was defined as the ratio of an absolute number of platelets to lymphocytes in peripheral blood before NACT. Similarly, NLR was defined as the ratio of neutrophils to lymphocytes. Tumor size before NACT was determined by US and/or MRI and was staged according to the eighth edition of the American Joint Committee on Cancer (AJCC). Clinical axillary LN negativity was defined as no abnormal LNs on ultrasound or MRI or confirmed negative by CNB. However, positive axillary LNs must be confirmed by CNB.

### Study design and development of the nomogram

**Figure 1:**
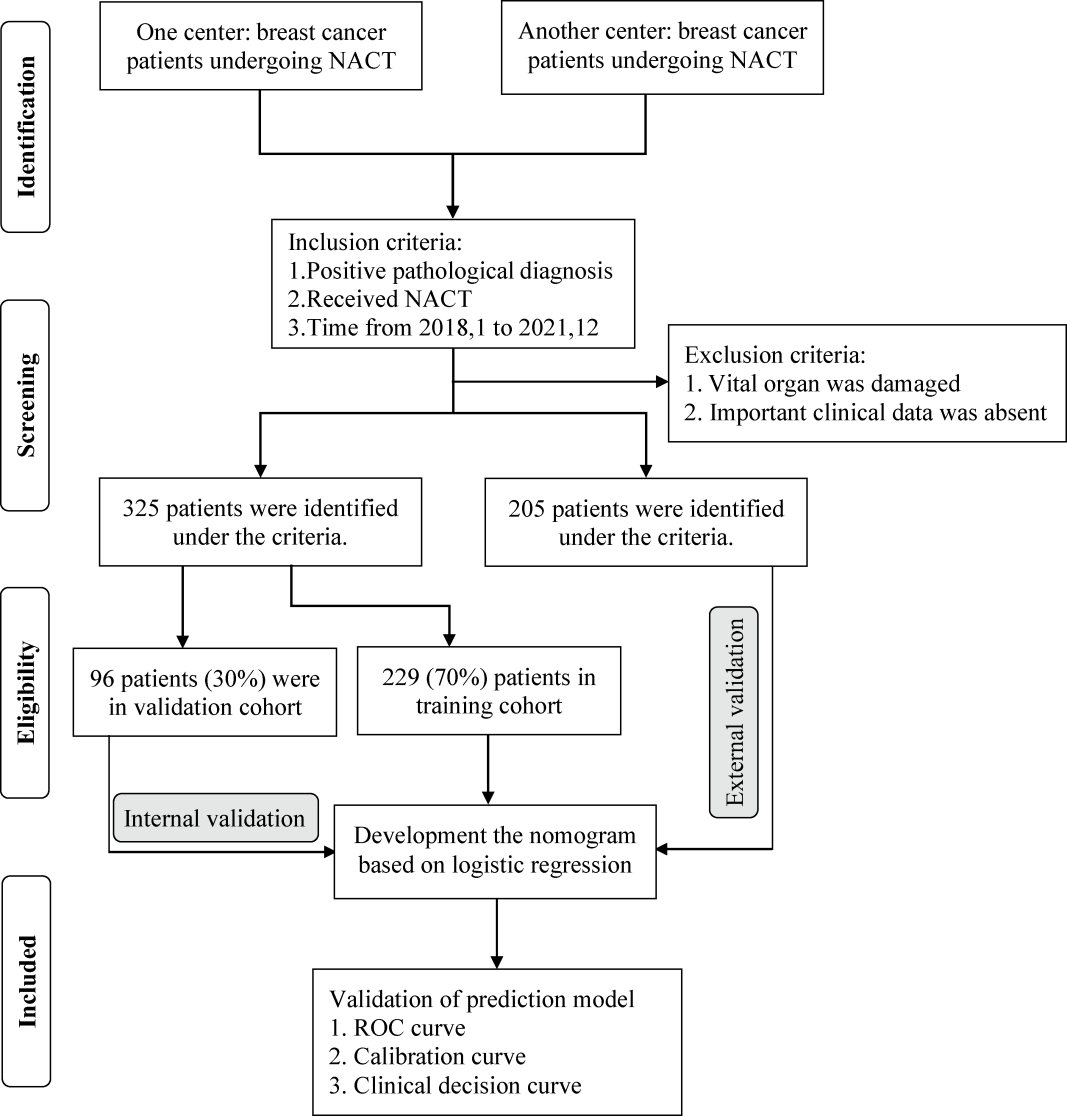
The strategy for selecting patients and flow diagram for developing and validating the prediction model.

The primary cohort consisted of 325 divided into the training and internal validation cohorts with a ratio of 7:3 using the R studio (version 4.0.3, http://www.r-project.org) function ‘caret’ to ensure that outcome events were distributed randomly between the two cohorts. Based on the training cohort, univariate logistic regression was used to preliminarily determine the potential relationship between each parameter and pCR. Subsequently, parameters with a p-value <0.05 in univariate analysis were further analyzed by multivariate logistic regression to select the most useful parameter for predicting pCR. The odds ratio (ORs) and corresponding 95% confidence intervals (CIs) were calculated. According to the regression coefficients of each screened factor in the multivariate analysis, the nomogram was visualized to quantitatively predict the probability of pCR for each BC patient treated with NACT. The internal validation cohort was applied to validate the efficiency and stability of the prediction model. The cohort with 205 BC patients from another institution was used as the external validation cohort to further test the prediction model; hence, it was also called the testing cohort. The flow diagram for developing and validating the prediction model is demonstrated in **Figure 1**.

### Validation of nomogram

The performance of the nomogram was evaluated with respect to its discrimination, calibration, and clinical usefulness based on the training cohort, internal validation cohort, and testing cohort. The discrimination and predictive accuracy of the nomogram was quantized to the area under the receiver operating characteristic (ROC) curve (AUC) by the package of ‘pROC’ in R studio. The value of AUC ranged from 0.5 to 1.0, and close to 1.0 meant higher discrimination and accuracy. Typically, a value greater than 0.7 indicates a reasonable estimation (15). Meanwhile, calibration curves based on 1,000 bootstrap resamples were plotted to assess the calibration of the nomogram. It showed the correlation between the predicted and observed probabilities. Therefore, the closer the calibration curve was to the straight line with a slope of 1, the better the predictive ability of the nomogram model. In addition, decision curve analysis (DCA) was applied to quantify the net benefit under different threshold probabilities to evaluate the clinical usefulness of the nomogram.

### Statistical analysis

Continuous variables conforming to normal distribution were presented as the mean and standard deviation (SD) and as the median and interquartile range (IQR) for non-normally distributed. Categorical variables were shown as frequencies and their percentage (%). Student’s t-tests (normally distributed) or Mann–Whitney U test (non-normally distributed) were applied to analyze the statistical difference of continuous variables. Categorical variables were analyzed by Fisher’s exact test or chi-square test. Univariate and multivariate logistic regression analyses were performed to select the most useful predictors from the training cohort. A two-sided p-value <0.05 was considered statistically significant. All statistical analyses and visualization were performed by using R studio statistical software version 4.0.3 (https://www.r-project.org).

### Ethics statement and informed consent

This study was exempt from the approval processes of the Institutional Review Boards because no personal information about patients was sought, and their identity would not be revealed in any publication. Written consent for publication was obtained from patients for data about the patients themselves.

## Results

### Demographics and clinicopathological characteristics of patients

The baseline clinicopathological characteristics of BC patients treated with NACT in the primary cohort are concluded in **Table 1**. Meanwhile, the clinicopathological features of the internal validation and training cohorts are presented in **Table 2**, which consisted of a random split of the primary cohort in a ratio of 7 to 3. The average age of the population was 48.2 years, of whom 85.2% were aged >35 years and 14.8% were aged ≤35 years, and the mean body mass index (BMI) was 23.2, of whom about 44.9% were postmenopausal and 91.1% completed all chemotherapy cycles. All 325 patients were divided into two groups according to whether pCR was obtained or not. In this cohort, a total of 126 obtained pCR, accounting for 38.8%. Overall, compared with group non-pCR, patients in group pCR had the following significant characteristics: older age, lower CA153 in the peripheral blood, smaller tumor diameter and lower stage, higher Ki67 index, higher proportion of HER2-positive tumors, and lower percentage of HR-positive tumors (all the p-value <0.05). In terms of molecular subtype, HER2-enriched patients have the highest pCR rate, followed by Luminal B/HER2-positive type and Triple-negative type, while Luminal B/HER2-negative type and Luminal A type had the lowest. Additionally, baseline data of 205 patients in the external validation cohort are presented in **Table 3**. A higher proportion of patients in the pCR group were older than 35 years, although this difference was not statistically significant at a p threshold of less than 0.05. Consistent with the primary cohort, patients receiving pCR in the external validation cohort also showed statistical differences in tumor size, Ki67 index, HER2 status, and HR status as compared with non-pCR patients (all the p-values <0.05). Similarly, patients with subtype HER2-enriched, Luminal B/HER2-positive, and triple-negative obtained higher pCR rates, while patients with subtype Luminal B/HER2-negative and Luminal A had lower rates.

**Table 1:** Clinicopathological characteristics of BC patients treated with NACT in the primary cohort.

Characteristics	Level	Overall	Non-pCR	pCR	p-Value
N (%)		325 (100)	199 (61.2)	126 (38.8)	
Age (mean (SD))		48.2 (10.8)	47.3 (11.4)	49.6 (9.6)	0.058
Age group (%)	>35	277 (85.2)	160 (80.4)	117 (92.9)	0.003
	≤35	48 (14.8)	39 (19.6)	9 (7.1)	
Menstruation (%)	Yes	179 (55.1)	114 (57.3)	65 (51.6)	0.372
	No	146 (44.9)	85 (42.7)	61 (48.4)	
BMI (median [IQR])		23.2 [21.1, 25.6]	23.4 [21.0, 25.9]	23.0 [21.2, 25.3]	0.687
PLR (median [IQR])		152.3 [117.0, 193.0]	154.6 [115.3, 193.7]	151.8 [121.8, 186.0]	0.935
NLR (median [IQR])		2.2 [1.7, 3.0]	2.3 [1.7, 2.9]	2.1 [1.6, 3.1]	0.597
Albumin (mean (SD))		42.9 (3.5)	43.0 (3.6)	42.7 (3.3)	0.478
CA125 (median [IQR])		13.7 [8.9, 18.3]	13.5 [8.5, 24.3]	14.3 [9.8, 17.0]	0.790
CA199 (median [IQR])		8.8 [4.0, 17.6]	9.3 [4.1, 17.6]	7.2 [3.1, 14.5]	0.282
CEA (median [IQR])		2.0 [1.3, 3.2]	2.15 [1.4, 3.8]	1.7 [1.1, 2.6]	0.153
CA153 (median [IQR])		12.6 [9.2, 21.2]	17.7 [10.4, 25.2]	10.3 [8.7, 15.4]	0.004
Tumor size (median [IQR])		3.6 [2.8, 4.9]	3.8 [3.0, 5.0]	3.1 [2.3, 4.0]	<0.001
AJCC_T (%)	T1	37 (11.4)	15 (7.5)	22 (17.6)	<0.001
	T2	224 (69.1)	132 (66.3)	92 (73.6)	
	T3	51 (15.7)	42 (21.1)	9 (7.2)	
	T4	12 (3.7)	10 (5.0)	2 (1.6)	
Axillary LN positive (%)	Yes	300 (92.3)	185 (93.0)	115 (91.3)	0.73
	No	25 (7.7)	14 (7.0)	11 (8.7)	
Complete NACT (%)	Yes	296 (91.1)	177 (88.9)	119 (94.4)	0.135
	No	29 (8.9)	22 (11.1)	7 (5.6)	
Pre_Ki67 (median [IQR])		0.3 [0.2, 0.5]	0.3 [0.2, 0.5]	0.4 [0.3, 0.6]	0.005
HER2 (%)	Positive	166 (51.1)	78 (39.2)	88 (69.8)	<0.001
	Negative	159 (48.9)	121 (60.8)	38 (30.2)	
HR (%)	Positive	173 (53.2)	131 (65.8)	42 (33.3)	<0.001
	Negative	152 (46.8)	68 (34.2)	84 (66.7)	
Histologic grade (%)	Unknown	53 (16.3)	15 (7.5)	38 (30.2)	<0.001
	I	2 (0.6)	2 (1.0)	0 (0.0)	
	II	122 (37.5)	89 (44.7)	33 (26.2)	
	III	148 (45.5)	93 (46.7)	55 (43.7)	
Molecular subtype (%)	Luminal A	23 (7.1)	22 (11.1)	1 (0.8)	<0.001
	Luminal B (HER2+)	78 (24.0)	48 (24.1)	30 (23.8)	
	Luminal B (HER2−)	72 (22.2)	60 (30.2)	12 (9.5)	
	HER2+	87 (26.8)	30 (15.1)	57 (45.2)	
	TNBC	65 (20.0)	39 (19.6)	26 (20.6)	

BC, breast cancer; pCR, pathologic complete response; N, number; SD, standard deviation; IQR, interquartile range; PLR, platelet-to-lymphocyte ratio; NLR, neutrophil-to-lymphocyte ratio; AJCC, American Joint Committee on Cancer; LN, lymph node; Pre_Ki67, Ki67 index before NACT; NACT, neoadjuvant chemotherapy; HER2, human epidermal growth factor receptor-2; HR, hormone receptor; TNBC, triple-negative breast cancer.

**Table 2:** Clinicopathological characteristics of BC patients treated with NACT in the training and internal validation cohorts.

Characteristics	Level	Training cohort				Validation cohort		
		Non-pCR	pCR	p-Value		Non-pCR	pCR	p-Value
N (%)		139	90			60	36	
Age (mean (SD))		47.5 (11.2)	50.4 (9.8)	0.046		46.9 (12.0)	47.8 (8.9)	0.702
Age group (%)	>35	113 (81.3)	83 (92.2)	0.035		47 (78.3)	34 (94.4)	0.07
	≤35	26 (18.7)	7 (7.8)			13 (21.7)	2 (5.6)	
Menstruation (%)	Yes	82 (59.0)	43 (47.8)	0.126		32 (53.3)	22 (61.1)	0.595
	No	57 (41.0)	47 (52.2)			28 (46.7)	14 (38.9)	
BMI (median [IQR])		23.4 [21.1, 25.8]	23.2 [21.2, 25.6]	0.963		23.4 [20.7, 26.8]	22.6 [21.2, 24.3]	0.644
PLR (median [IQR])		156.7 [113.5, 195.8]	158.4 [125.3, 205.9]	0.486		144.5 [131.2, 192.2]	129.8 [106.6, 172.4]	0.131
NLR (median [IQR])		2.40 [1.7, 3.3]	2.1 [1.7, 3.1]	0.533		2.2 [1.7, 2.7]	2.1 [1.5, 3.1]	0.958
Albumin (mean (SD))		43.2 (3.6)	42.5 (3.5)	0.269		42.4 (3.7)	42.9 (3.1)	0.631
CA125 (median [IQR])		13.4 [8.3, 21.0]	12.1 [9.7, 17.0]	0.956		15.8 [9.9, 36.4]	14.4 [11.3, 16.9]	0.550
CA199 (median [IQR])		9.2 [4.2, 17.6]	6.8 [3.6, 15.2]	0.473		9.8 [4.2, 38.2]	8.3 [3.4, 13.4]	0.334
CEA (median [IQR])		2.3 [1.5, 4.1]	1.6 [1.1, 2.6]	0.062		1.75 [1.3, 2.6]	2.2 [1.3, 2.7]	0.678
CA153 (median [IQR])		16.2 [10.4, 25.9]	10.4 [9.2, 17.8]	0.062		18.0 [12.3, 23.5]	9.7 [7.1, 12.5]	0.020
Tumor size (median [IQR])		3.9 [3.0, 5.0]	3.0 [2.2, 4.0]	<0.001		3.5 [2.8, 5.0]	3.3 [2.5, 4.5]	0.511
AJCC_T (%)	T1	7 (5.0)	16 (18.0)	<0.001		8 (13.3)	6 (17.6)	0.435
	T2	93 (66.9)	66 (74.2)			38 (63.3)	24 (70.6)	
	T3	32 (23.0)	5 (5.6)			11 (18.3)	4 (11.8)	
	T4	7 (5.0)	2 (2.2)			3 (5.0)	0 (0.0)	
Axillary LN positive (%)	Yes	131 (94.2)	83 (92.2)	0.741		54 (90.0)	32 (88.9)	1
	No	8 (5.8)	7 (7.8)			6 (10.0)	4 (11.1)	
Complete NACT (%)	Yes	125 (89.9)	85 (94.4)	0.335		52 (86.7)	34 (94.4)	0.388
	No	14 (10.1)	5 (5.6)			8 (13.3)	2 (5.6)	
Pre_Ki67 (median [IQR])		0.3 [0.2, 0.5]	0.4 [0.3, 0.6]	0.008		0.3 [0.2, 0.5]	0.4 [0.3, 0.6]	0.302
HER2 (%)	Positive	51 (36.7)	63 (70.0)	<0.001		27 (45.0)	25 (69.4)	0.034
	Negative	88 (63.3)	27 (30.0)			33 (55.0)	11 (30.6)	
HR (%)	Positive	95 (68.3)	28 (31.1)	<0.001		36 (60.0)	14 (38.9)	0.073
	Negative	44 (31.7)	62 (68.9)			24 (40.0)	22 (61.1)	
Histologic grade (%)	I	/	/			2 (3.6)	0 (0.0)	0.604
	II	67 (51.9)	24 (37.5)	0.082		22 (40.0)	9 (37.5)	
	III	62 (48.1)	40 (62.5)			31 (56.4)	15 (62.5)	
Molecular subtype (%)	Luminal A	18 (12.9)	1 (1.1)	<0.001		4 (6.7)	0 (0.0)	0.053
	Luminal B (HER2+)	33(23.7)	22 (24.4)			15 (25.0)	8 (22.2)	
	Luminal B (HER2−)	43(30.9)	5 (5.6)			17 (28.3)	7 (19.4)	
	HER2+	19 (13.7)	41 (45.6)			11 (18.3)	16 (44.4)	
	TNBC	26 (18.7)	21 (23.3)			13 (21.7)	5 (13.9)	

BC, breast cancer; pCR, pathologic complete response; N, number; SD, standard deviation; IQR, interquartile range; PLR, platelet-to-lymphocyte ratio; NLR, neutrophil-to-lymphocyte ratio; AJCC, American Joint Committee on Cancer; LN, lymph node; Pre_Ki67, Ki67 index before NACT; NACT, neoadjuvant chemotherapy; HER2, human epidermal growth factor receptor-2; HR, hormone receptor; TNBC, triple-negative breast cancer.

**Table:** Table 3. Clinicopathological characteristics of the test cohort of BC patients treated with NACT in the testing cohort.

Characteristics	Level	Overall	Non-pCR	pCR	p-Value
N (%)		205 (100)	163 (79.5)	42 (20.5)	
					
Age [mean (SD)]		47.9 (10.4)	48.0 (10.9)	47.6 (8.4)	0.851
Age group (%)	>35	167 (81.5)	128 (78.5)	39 (92.9)	0.056
	≤35	38 (18.5)	35 (21.5)	3 (7.1)	
Menstruation (%)	Yes	116 (56.6)	92 (56.4)	24 (57.1)	1
	No	89 (43.4)	71 (43.6)	18 (42.9)	
BMI (median [IQR])		23.5 [21.5, 26.4]	23.5 [21.6, 26.6]	23.4 [21.2, 25.8]	0.609
PLR (median [IQR])		132.8 [102.1, 167.4]	131.3 [100.7, 163.4]	139.1 [105.4, 170.3]	0.484
NLR (median [IQR])		2.1 [1.6, 2.8]	2.1 [1.6, 2.8]	2.2 [1.7, 2.8]	0.801
Albumin (mean (SD))		44.7 (2.9)	44.7 (2.9)	44.6 (2.9)	0.783
CA125 (median [IQR])		11.8 [8.6, 21.5]	10.9 [7.8, 21.1]	14.8 [10.0, 24.3]	0.052
CA199 (median [IQR])		10.8 [7.4, 15.2]	11.0 [7.3, 14.7]	10.6 [8.4, 17.8]	0.507
CEA (median [IQR])		1.6 [1.1, 2.5]	1.5 [1.1, 2.4]	1.7 [1.2, 2.6]	0.511
CA153 (median [IQR])		10.3 [6.9, 17.3]	10.2 [6.9, 18.0]	10.3 [7.0, 14.9]	0.935
Tumor size (median [IQR])		3.5 [2.5, 4.7]	3.7 [2.8, 4.9]	2.9 [2.1, 4.0]	0.001
AJCC_T (%)	T1	24 (11.7)	14 (8.6)	10 (23.8)	0.005
	T2	131 (63.9)	103 (63.2)	28 (66.7)	
	T3	31 (15.1)	27 (16.6)	4 (9.5)	
	T4	19 (9.3)	19 (11.7)	0 (0.0)	
Axillary LN positive (%)	Yes	167 (81.5)	134 (82.2)	33 (78.6)	0.750
	No	38 (18.5)	29 (17.8)	9 (21.4)	
Complete NACT (%)	Yes	123 (60.0)	103 (63.2)	20 (47.6)	0.097
	No	82 (40.0)	60 (36.8)	22 (52.4)	
Pre_Ki67 (median [IQR])		0.3 [0.2, 0.5]	0.3 [0.2, 0.5]	0.4 [0.3, 0.6]	0.005
HER2 (%)	Positive	74 (36.1)	51 (31.3)	23 (54.8)	0.008
	Negative	131 (63.9)	112 (68.7)	19 (45.2)	
HR (%)	Positive	135 (65.9)	115 (70.6)	20 (47.6)	0.009
	Negative	70 (34.1)	48 (29.4)	22 (52.4)	
Histologic grade (%)	Unknown	29 (14.1)	24 (14.7)	5 (11.9)	0.716
	I	1 (0.5)	1 (0.6)	0 (0.0)	
	II	133 (64.9)	107 (65.6)	26 (61.9)	
	III	42 (20.5)	31 (19.0)	11 (26.2)	
Molecular subtype (%)	Luminal A	23 (11.2)	23 (14.1)	0 (0.0)	0.002
	Luminal B (HER2+)	44 (21.5)	32 (19.6)	12 (28.6)	
	Luminal B (HER2−)	69 (33.7)	61 (37.4)	8 (19.0)	
	HER2+	29 (14.1)	18 (11.0)	11 (26.2)	
	TNBC	40 (19.5)	29 (17.8)	11 (26.2)	

BC, breast cancer; pCR, pathologic complete response; N, number; SD, standard deviation; IQR, interquartile range; PLR, platelet-to-lymphocyte ratio; NLR, neutrophil-to-lymphocyte ratio; AJCC, American Joint Committee on Cancer; LN, lymph node; Pre_Ki67, Ki67 index before NACT; NACT, neoadjuvant chemotherapy; HER2, human epidermal growth factor receptor-2; HR, hormone receptor; TNBC, triple-negative breast cancer.

### Univariate and multivariate analyses of factors associated with the pathological complete response

**Figure 2:**
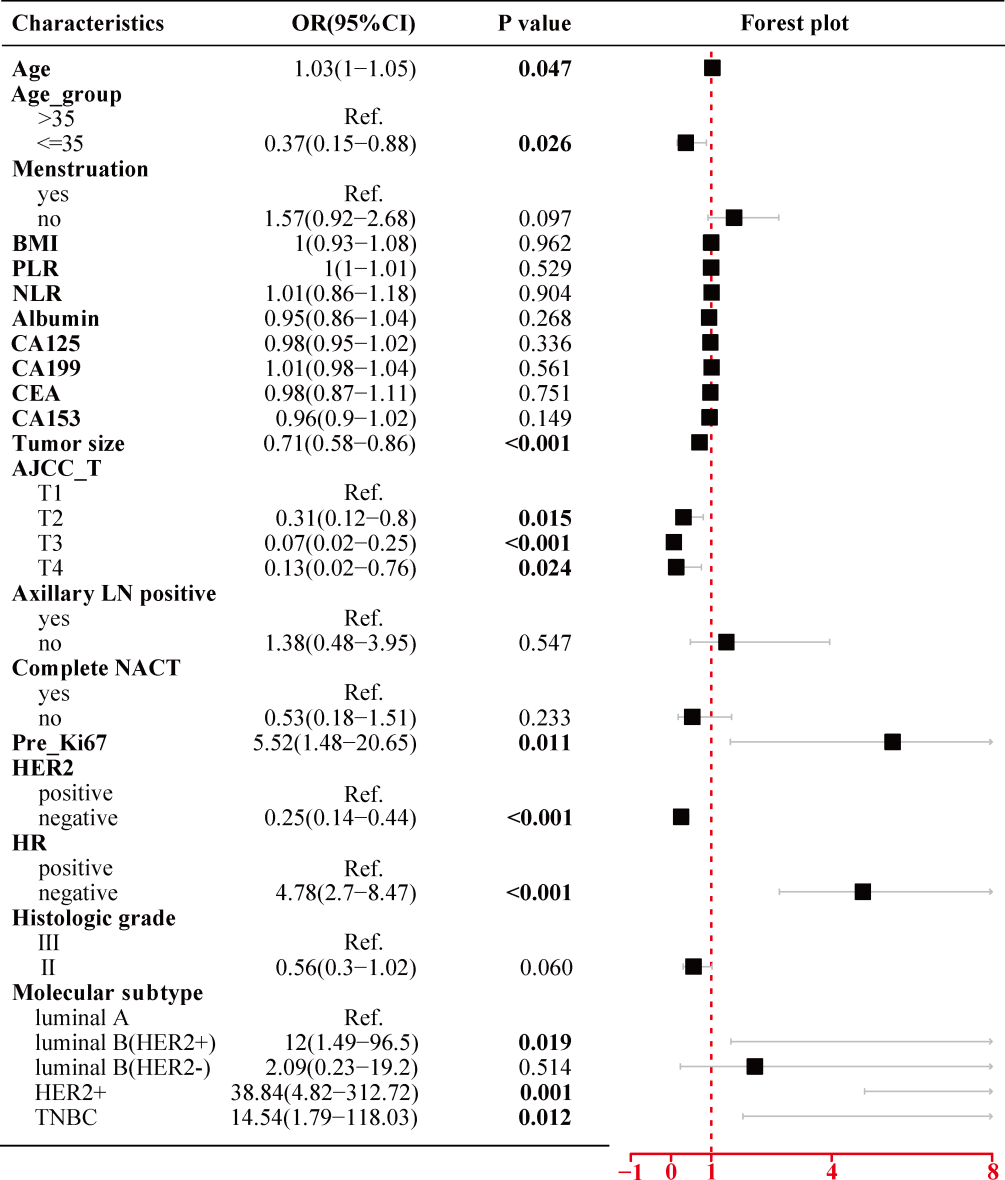
Univariate logistic regression analysis of the clinicopathological parameters for pCR in BC patients treated with NACT using the training cohort. pCR, pathological complete response; BC, breast cancer; NACT, neoadjuvant chemotherapy; BMI, body mass index; PLR, platelet-to-lymphocyte ratio; NLR, neutrophil-to-lymphocyte ratio; AJCC, American Joint Committee on Cancer; LN, lymph node; NACT, neoadjuvant chemotherapy; Pre_Ki67, Ki67 index before NACT; HER2, human epidermal growth factor receptor 2; HR, hormone receptor.

**Figure 3:**
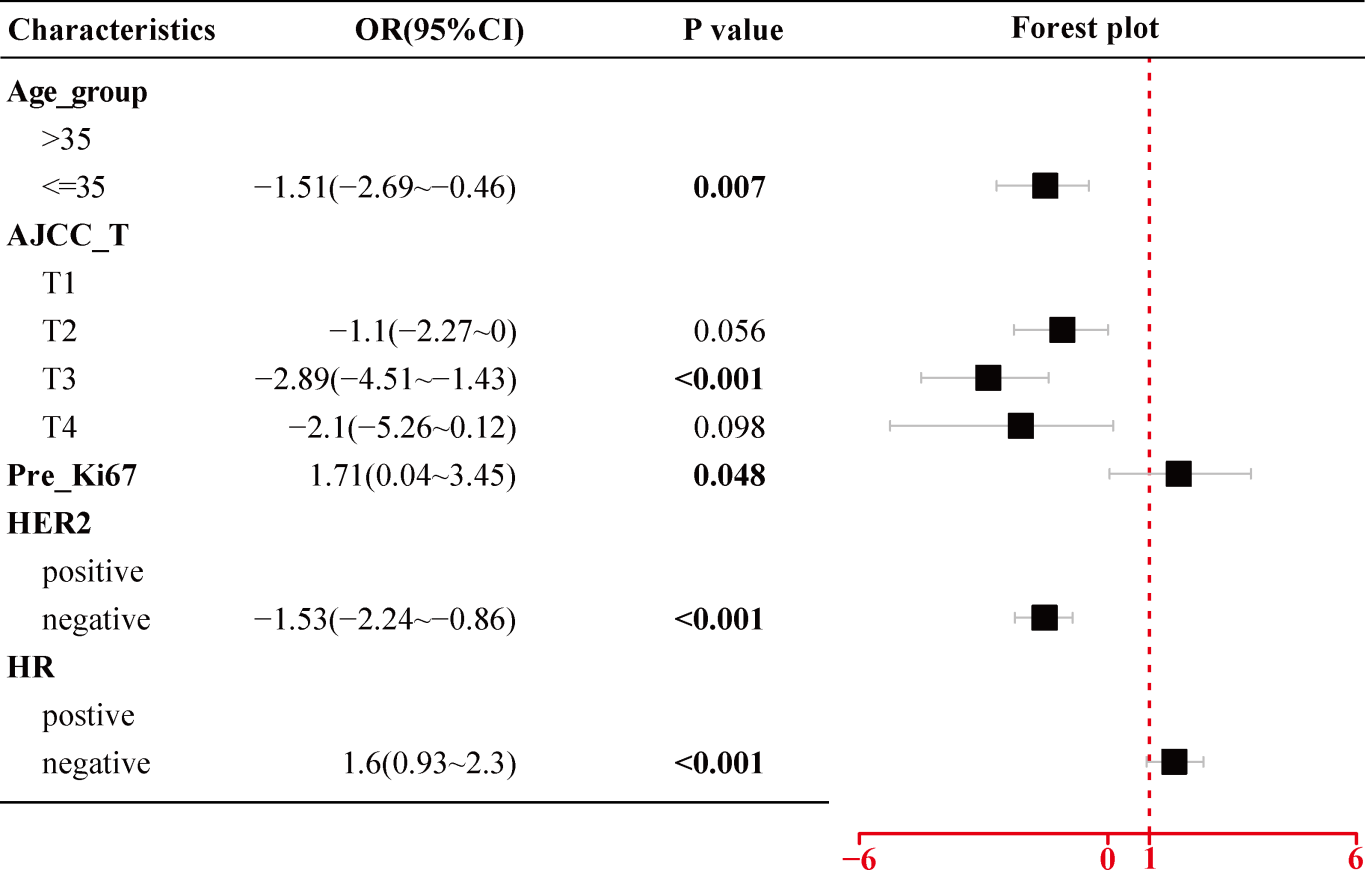
Multivariate logistic regression analysis of the selected clinicopathological parameters for pCR in BC patients treated with NACT using the training cohort. pCR, pathological complete response; BC, breast cancer; NACT, neoadjuvant chemotherapy; AJCC, American Joint Committee on Cancer; Pre_Ki67, Ki67 index before NACT; HER2, human epidermal growth factor receptor 2; HR, hormone receptor.

As shown in **Figure 2**, the univariate logistic regression analysis in the training cohort suggested that eight factors including age (p = 0.047), age group (p = 0.026), tumor size (p < 0.001), AJCC_T stage, Ki67 index before NACT (Pre_Ki67, p = 0.011), HER2 status (p < 0.001), HR status (p < 0.001), and molecular subtype were associated with the pCR of NACT. Due to the potential overlap between age and age group, tumor size and AJCC_T stage, molecular subtypes, and markers (Ki67, HER2, and HR), only one of each was further included in the multivariate analysis. Further multivariate logistic regression analysis (**Figure 3**) showed that five factors including age group (≤35: OR, −1.51; 95% CI, −2.69 to −0.46; p = 0.007), AJCC_T (T2: OR, −1.1; 95% CI, −2.27~0; p = 0.056; T3: OR, −2.89; 95% CI, −4.51 to −1.43; p < 0.001; T4: OR, −2.1; 95% CI, −5.26 to −0.12; p = 0.098), Pre_Ki67 (OR, 1.71; 95% CI, 0.04~3.45; p = 0.048), HER2 status (negative: OR, −1.53; 95% CI, −2.24 to −0.86; p < 0.001), and HR (negative: OR, 1.6; 95% CI, 0.93~2.3; p < 0.001) status were independent predictors for pCR of NACT.

### Development of nomogram prediction model

**Figure 4:**
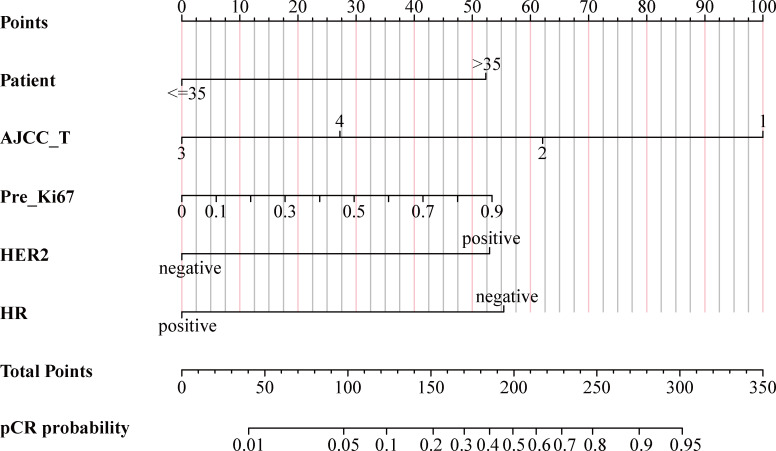
Nomogram for predicting probabilities of pCR in BC patients treated with NACT. pCR, pathological complete response; BC, breast cancer; NACT, neoadjuvant chemotherapy; AJCC, American Joint Committee on Cancer; Pre_Ki67, Ki67 index before NACT; HER2, human epidermal growth factor receptor 2; HR, hormone receptor.

As shown in **Figure 4**, using the regression coefficients of multivariate logistic regression models to weight each feature in our models, the nomogram based on the above five independent predictors was developed to quantitatively predict the probability of pCR for each BC patient treated with NACT. Each value of each included variable was assigned a score accordingly on the points scale. The scores for each variable were added to the model to get the total score. The predictions corresponding to the total score contribute to the prediction of the pCR probability of each patient.

### Validation of nomogram prediction model

#### Internal validation

**Figure 5:**
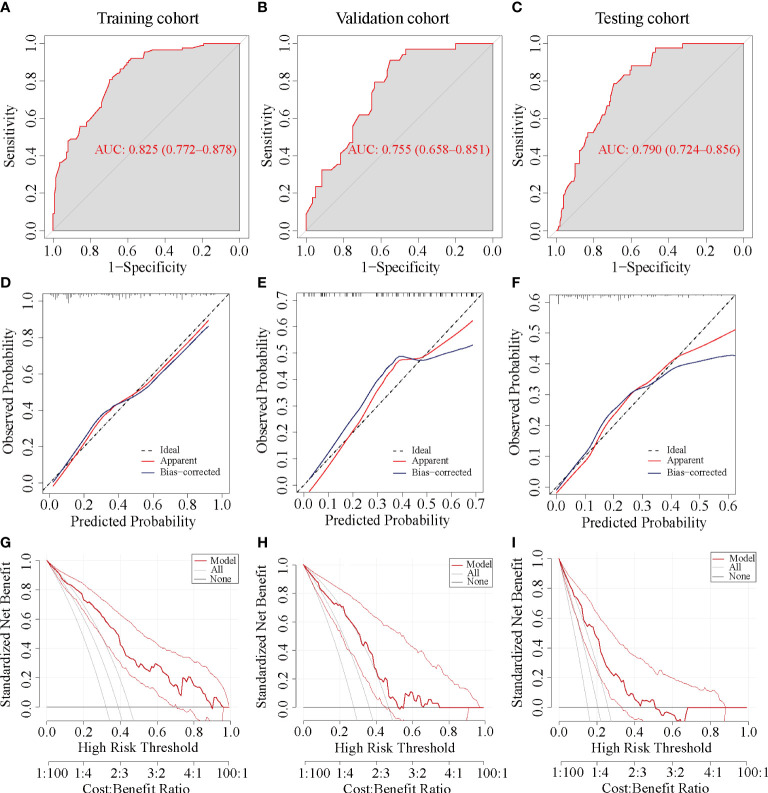
ROC, calibration, and DCA curve of the nomogram prediction models in each cohort. ROC, receiver operating characteristic; AUC, area under the ROC curve; DCA, decision curve analysis. **(A, D, G)** ROC, calibration, and DCA curve of the nomogram prediction model in the training cohort, respectively. **(B, E, H)** ROC, calibration, and DCA curve of the nomogram prediction model in the internal validation cohort. **(C, F, I)** ROC, calibration, and DCA curve of the nomogram prediction model in the testing cohort.

The AUC values of the ROC curves were 0.825 (95% CI, 0.772~0.878) in the training cohort and 0.755 (95% CI, 0.658~0.851) in the internal cohort, which were greater than 0.7, reflecting the good accuracy and discrimination of the model (**Figures 5A, B**). The calibration curve of the nomogram for the probability of pCR showed favorable consistency between prediction and observation in both the training and internal cohorts (**Figures 5D, E**). The DCA for the nomogram is presented in **Figures 5G, H**, which indicated that when the threshold probabilities of the training and internal cohorts were in the range of 0%~90% and 0%–55%, respectively, a higher net clinical benefit could be achieved than the hypothetical non-testing or all testing scenarios.

#### External validation

The ROC curve yielded an AUC of 0.79 (95% CI, 0.724 to 0.856) in the external validation cohort (**Figure 5C**). Consistent with the training and internal validation cohorts, the calibration curve for the testing cohort was also significantly closer to the 45° diagonal, implying a good calibration of the model (**Figure 5F**). The DCA curve of the nomogram in the testing cohort presented a favorable net benefit when the threshold probability was 0%~50% (**Figure 5I**).

## Discussion

Predicting the probability of pCR contributes to evaluating the benefit of NACT in newly diagnosed BC patients and assists in selecting the optimal surgical approach before surgery. However, there is still a lack of consensus on imaging-based assessment of pathological response after NACT in BC patients, and pCR cannot be reliably predicted (10, 16). Thus, in this study, 325 patients in one center were randomly divided into the training and validation cohorts in a ratio of 7 to 3, and a nomogram prediction model based on clinicopathological characteristics was constructed and internally validated. Meanwhile, 205 patients from another center were used as the testing cohort to further externally validate the performance of the predictive model.

This study confirmed that HER-2 overexpression, Luminal B HER2+, and triple-negative breast cancer (TNBC) were favorable subtypes to achieve a pCR, while the pCR rates of Luminal B/HER2− and Luminal A type were low, which was consistent with the majority of previously published studies (6, 17–19). One explanation was that luminal BCs were slowly proliferating tumors that were more amenable to local therapy and benefited from longer endocrine therapy, while HER2-amplified BC and TNBC were rapidly proliferating tumors that were sensitive to NACT (20). Therefore, whether in univariate or multivariate logistic analysis, HR status, HER2 status, and Ki67 index before NACT related to molecular subtyping were the independent predictors of pCR. A higher pCR rate could be achieved in patients with negative or low expressed ER, positive or overexpressed HER2, and a higher Ki67 index before NACT. Moreover, a previous study had confirmed that patients with ≤10% ER expression had an extremely high possibility (31.3%) of achieving total pCR; in comparison, the pCR rate of the other patients was only 8.9% by classifying the expression of ER into five levels (21). The index of Ki67 before NACT was considered to reflect the ability of tumor cell proliferation and closely related to the sensitivity to the NACT (22). Thus, the Ki67 index was consistently recognized as an independent predictor of NACT response (23, 24). As for HER2, thanks to the use of trastuzumab and/or pertuzumab, the efficacy of NACT in HER2-positive patients had been greatly improved, which also made HER2 an independent predictor of the efficacy of NACT (25, 26). The pCR rates of the two institutions were 38.8% and 20.5%, respectively, which were consistent with the range reported in the literature (27–29).

The correlations between the tumor size and NACT efficacy have remained controversial. In this study, we concluded that smaller tumor size or lower AJCC T staging was associated with a higher pCR rate, which was consistent with other studies. Goorts et al. made it clear that lower T stages had significantly higher pCR rates than higher T stages, and the clinical tumor stage was the most important predictor of pCR rate after NACT in BC patients (30). Another study confirmed that tumors >5 cm were associated with a lower likelihood of having a pCR following NAC even when accounting for receptor status (31). However, a different view suggested that tumor size on the probability of pCR was not statistically significant in any of the molecular subtypes (32). Another study found a correlation between tumor size and pCR in univariate analysis but disappeared in multivariate analysis (33). The mean age of patients in the pCR group was higher than that of the non-pCR group (49.6 *vs*. 47.3), and the pCR rate was lower in BC patients younger than 35 years. This result could be explained that younger BC patients in East Asia were characterized by a high prevalence of Luminal A subtype and a low prevalence of basal-like subtype (34, 35). The clinicopathological characteristics and age-specific incidences between East Asian and American women had been proved to be racially different (36). Among American women, the proportion of ER positivity increased gradually with age, while the proportion of HER2+ and TNBC decreased with age (37, 38). The breast tumors in younger women aged <35 were thought to be more aggressive, with larger tumor size, more positive LNs, poorer differentiation, and a lack of HR (34). Additionally, the pathological diagnostic criteria for ER and PR positivity may vary between countries or hospitals (39).

There were several other factors that may affect the efficacy of NACT. Neither PLR nor NLR in this study showed a predictive value for pCR, although the study by Graziano et al. confirmed low levels of both NLR (≤2.42) and PLR (≤104.72), which indicated a status of immune system activation that predict pCR in BC patients treated with NACT (40). It could be interpreted that the levels of inflammatory cells in the body were unstable due to many factors, which made it inaccurate in predicting the efficacy of NACT. There were also reports in the literature that increased tumor-infiltrating lymphocyte (TIL) levels could predict pathological response in all BC subtypes and were associated with a survival benefit in the TNBC and HER2+ but not the luminal-HER2− subtype (41). The value of PLR and NLR in predicting the efficacy of neoadjuvant chemotherapy for other tumors had been widely reported (42, 43). In addition, although the non-PCR group had a higher BMI than the PCR group, which seemed to be consistent with previous reports (44), the difference was not statistically significant. Previous studies have indicated that obesity is an independent prognostic factor of decreased pCR to NACT in BC patients (45, 46). However, Del Prete et al. also reported that obesity does not correlate with pathological responses in their patients’ series (47). Alan et al. concluded that BMI was not a predictive biomarker for pCR, which was similar to the result of our study (48). Furthermore, our study confirmed that tumor makers, axillary LN positive, menstrual status before NACT (49), and albumin levels (50) did not show significant values in predicting PCR, which was consistent with previous studies.

There were several limitations that should be acknowledged. Firstly, due to the variety of regimens and the limited sample size, chemotherapy regimens had not been analyzed, which was an important factor affecting pCR. Secondly, the relatively small sample size and regional variations in the level of medical service may weaken the result. Finally, as a retrospective study, there were inherent selection biases and uncontrollable confounding factors. Therefore, the efficacy of the nomogram needed to be further verified by multicenter large-sample randomized controlled experiments.

## Conclusion

This study constructed a validated nomogram based on age, AJCC T stage, Ki67 index before NACT, HER2, and HR status, which can be non-invasively applied to personalize the prediction of pCR in BC patients treated with NACT both before NACT and before surgery. The nomogram has the potential to assist clinicians in screening BC patients for NACT and adjusting the optimal surgical approach for BC patients after NACT.

## Data availability statement

The raw data supporting the conclusions of this article will be made available by the authors, without undue reservation.

## Ethics statement

Ethical review and approval was not required for the study on human participants in accordance with the local legislation and institutional requirements. The patients/participants provided their written informed consent to participate in this study.

## Author contributions

Conception and design: all authors. Administrative support: XQ, MR, and SZ. Collection and assembly of data: BQ, JY, and JZ. Data analysis and interpretation: BQ, JY, and LH. Manuscript writing: BQ. Critical revision of the manuscript: JZ and SZ. All authors contributed to the article and approved the submitted version.

## Conflict of interest

The authors declare that the research was conducted in the absence of any commercial or financial relationships that could be construed as a potential conflict of interest.

## Publisher’s note

All claims expressed in this article are solely those of the authors and do not necessarily represent those of their affiliated organizations, or those of the publisher, the editors and the reviewers. Any product that may be evaluated in this article, or claim that may be made by its manufacturer, is not guaranteed or endorsed by the publisher.
